# Comparative Evaluation of Real-Time PCR Methods for Human Noroviruses in Wastewater and Human Stool

**DOI:** 10.1371/journal.pone.0160825

**Published:** 2016-08-15

**Authors:** Yoshifumi Masago, Yoshimitsu Konta, Shinobu Kazama, Manami Inaba, Toshifumi Imagawa, Kentaro Tohma, Mayuko Saito, Akira Suzuki, Hitoshi Oshitani, Tatsuo Omura

**Affiliations:** 1 New Industry Creation Hatchery Center, Tohoku University, Sendai, Miyagi, Japan; 2 Graduate School of Medicine, Tohoku University, Sendai, Miyagi, Japan; Seconda Universita degli Studi di Napoli, ITALY

## Abstract

Selecting the best quantitative PCR assay is essential to detect human norovirus genome effectively from clinical and environmental samples because no cell lines have been developed to propagate this virus. The real-time PCR methods for noroviruses GI (4 assays) and GII (3 assays) were evaluated using wastewater (n = 70) and norovirus-positive stool (n = 77) samples collected in Japan between 2012 and 2013. Standard quantitative PCR assays recommended by the U.S. Environmental Protection Agency, International Organization for Standardization, and Ministry of Health, Labour and Welfare, Japan, together with recently reported assays were included. Significant differences in positive rates and quantification cycles were observed by non-parametric analysis. The present study identifies the best assay for norovirus GI and GII to amplify norovirus genomes efficiently.

## Introduction

Noroviruses (Family *Caliciviridae*, Genus *Norovirus*) are the leading etiologic agents causing viral gastroenteritis, with sporadic cases and outbreaks reported worldwide[1]. Because of its rapid molecular evolution, new genotypes and variants of the virus are reported frequently, and over 35 norovirus genotypes have been identified [2]. Accordingly, it is important to constantly ascertain the efficiency of the detection methods for the emerging genotypes.

Despite great efforts by researchers [3], no cell line is currently available to propagate human norovirus. Thus, molecular methods using PCR and quantitative real-time PCR (qPCR) are used widely to detect noroviruses in various fields including diagnostics, outbreak investigation, and environmental monitoring. Several qPCR methods have been developed and validated to detect noroviruses known to infect humans, namely genogroups I, II, and IV (GI, GII, and GIV). Some of them were included in standard protocols such as U.S. EPA Method 1615 [4], ISO/TS 15216–1 [5], and a standard protocol developed by the Ministry of Health, Labour and Welfare, Japan [6]. Standardization enabled many laboratories to detect noroviruses from stool, water, and food samples using validated effective methods. However, the sensitivity of the qPCR methods included in the standard protocols have not been evaluated, except for two reports comparing the European Committee on Normalisation (CEN) method included in ISO/TS 15216–1 and two commercial kits [7], or the CEN method and the new assay developed within the study [8]. For accurate quantification of norovirus genomes, selecting appropriate assays is required. For diagnostic purposes, qPCR methods are selected for high sensitivity and specificity. On the other hand, since environmental analysis mainly focuses on quantifying noroviruses in the samples, selecting sensitive qPCR assays is also a priority.

The aim of the present study was to compare the commonly used qPCR methods for norovirus GI and GII. For that purpose, the standard assays recommended by the U.S. EPA, ISO, and Ministry of Health, Labour and Welfare, Japan were selected. In addition, recent GI and GII assays reported to be more sensitive than the assays in ISO/TS 15216–1 [8] were also included. To evaluate these qPCR assays in practical situation, wastewater and stool samples collected in Japan between 2012 and 2013 were analyzed using the qPCR methods to cover environmental and clinical applications. Fecal samples were collected through a gastroenteritis surveillance program. They contain norovirus genotypes/variants that are prevalent or emerging at the time. Wastewater is known to contain multiple genotypes/variants of norovirus excreted from patients in the area, and the concentration of each genotype/variant is different. Non-parametric tests were conducted to compare the positive rates and quantification cycle (Cq) between the assays.

## Materials and Methods

### Wastewater samples

Wastewater samples were collected weekly at Matsushima Wastewater Treatment Plant in Japan, from August 2012 to December 2013. We obtained permission from the plant to collect the samples for this study. Primary effluent after primary sedimentation tank was collected, transported to the laboratory on ice, and stored in a freezer (−80°C) until they were concentrated by the polyethylene glycol precipitation method. Before the concentration process, 2.7 × 10^7^–1.0 × 10^9^ copies of the murine norovirus strain S7-PP3 provided by Prof. Yukinobu Tohya (Nihon University, Japan) was spiked into each sample as a process control. Then, polyethylene glycol 6000 (8% w/v final) (Wako Pure Chemical Industries, Osaka, Japan) and sodium chloride (2.3% w/v final) (Kanto Chemicals, Tokyo, Japan) were added to 40 mL of wastewater, and the mix was stored at 4°C overnight with gentle mixing. The coagulated particulate matter containing the virus particles was pelleted by centrifugation (9,000 × g; 30 min), and resuspended into 1 mL of deionized water. After vigorous vortexing, the released viral particles were separated by centrifugation (10,000 × g; 10 min), and the supernatant was collected as the virus concentrate. Viral RNA was extracted from the virus concentrates using the QIAamp Viral RNA Mini QIAcube Kit (Qiagen, Hilden, Germany) and QIAcube (Qiagen, Hilden, Germany). The cDNA was obtained using the iScript Advanced cDNA synthesis kit (Bio-Rad, Hercules, CA), following the manufacturers’ instructions.

### Stool samples

Stool samples were collected from November 2011 to July 2013 through our gastroenteritis surveillance program involving two pediatric clinics, an internal medicine and pediatric outpatient clinic, and a general hospital. Stools from gastroenteritis patients were collected using rectal swabs or a rapid test kit (BD Rota/Adeno Examan stick, Becton-Dickinson, New Jersey, USA) after obtaining informed consent. Since the participation (collecting fecal samples from the patients) has minimum risk and the procedure (etiological diagnostic test of acute gastroenteritis) is a part of the general process of diagnosis of the disease, we obtained verbal consent after explaining the study based on the consent form, and the consent was recorded in the patient’s medical chart. We obtained informed consent from the family or caretakers on behalf of the minors/children. The samples were transported to our laboratory on ice, and RNA extraction was performed immediately with the QIAmp Viral RNA Mini QIAcube kit (Qiagen, Hilden, Germany) and QIAcube (Qiagen, Hilden, Germany), followed by cDNA synthesis with SuperScript III Reverse Transcriptase (Life Technologies, Carlsbad, CA, USA), following the manufacturers’ instructions. Norovirus-positive stool samples were selected by qPCR using TaqMan Fast Advanced Master Mix (Life Technologies, Carlsbad, CA, USA) and Applied Biosystems 7500 Real-Time PCR System (Life Technologies, Carlsbad, CA, USA). The primers and probes used are listed as GI-A and GII-A assays in Table 1 [9]. The cDNA of the norovirus-positive stool samples was used in the subsequent analysis. This study was approved by the Research Ethics Committee of Tohoku University Graduate School of Medicine, Sendai, Japan.

**Table 1:** Quantitative polymerase chain reaction assays used in the evaluation.

Assay^a^	Forward primer	Reverse primer	TaqMan probe	PCR condition	Reference
Norovirus GI					
GI-A (Japan)	COG1F	COG1R	RING1(a)-TP and RING1(b)-TP	95°C, 15 s; 56°C, 1 min	[9]
GI-B (EPA)	JJV1F	JJV1R	JJV1P	95°C, 15 s; 60°C, 1 min	[10]
GI-C (EPA and ISO)	QNIF4	NV1LCR	NV1Cpr	95°C, 15 s; 60°C, 1 min	[11]
GI-D	NIFG1F	NV1LCR	NIFG1P	95°C, 15 s; 55°C, 1 min; 65°C, 1 min	[8]
Norovirus GII					
GII-A (Japan)	COG2F	COG2R	RING2-TP	95°C, 15 s; 56°C, 1 min	[9]
GII-B (EPA and ISO)	QNIF2d	COG2R	QNIFS	95°C, 15 s; 60°C, 1 min	[12]
GII-C	NIFG2F	COG2R	QNIFS	95°C, 15 s; 55°C, 1 min; 65°C, 1 min	[8]

^a^ The name of the standard protocols (EPA, ISO and Japan) using the assays were shown in parenthesis.

### Genotyping noroviruses in the samples

To confirm that noroviruses were present in the stool samples, the genotypes and variants were identified by amplification of the partial capsid protein (VP1) gene and RNA-dependent RNA polymerase gene of norovirus GI and GII by single-round PCR and/or nested PCR, and sequencing using the primers p290, COG1F, COG2F, G1SKR, and G2SKR [9,13,14]. The genotypes and variants were identified using the Norovirus Genotyping Tool [15]. In total, 77 norovirus-positive stool samples (5 GI and 72 GII) were used for the following analysis.

The genotypes of noroviruses in the wastewater samples were not investigated because it requires substantial efforts. Cloning-sequencing method [16–18] and pyrosequencing [19] are often used to identify genotypes in wastewater samples, but they are time- and labor-consuming and were not feasible to conduct only to confirm the presence of noroviruses in the samples.

### Quantification of norovirus cDNA for qPCR efficiency assessments

To evaluate the Cq values of the qPCR methods, the norovirus cDNA was quantified using SsoFast probes Supermix (Bio-Rad, Hercules, CA) and CFX96 (Bio-Rad, Hercules, CA). The primers and TaqMan probes used in the analysis are listed in Table 1. Each reaction mix (20 μL) contained 10 μL of the master mix, 5 μL of cDNA, the primers, and TaqMan probe at the concentrations specified in the references. To reduce inter-assay error, all qPCR assays used cDNA from the same reverse transcription (RT) reaction (i.e., cDNA from the same tube), and the cDNA was freeze-thawed once before being applied to each qPCR assays. PCR was initiated with a polymerase activation step (95°C; 10 min), followed by 50 cycles of amplification as shown in Table 1. The Cq values were determined using CFX Manager Software Ver. 2.1 (Bio-Rad, Hercules, CA). The measurement was not replicated in this study (one well was used for each sample), because although replicating measurements would enhance accuracy of the Cq values, replicating may produce elusive results (e.g. one positive and two negatives in triplicating), which is difficult to use in statistical analyses. The murine norovirus strain S7-PP3, spiked into the wastewater samples, was also quantified by qPCR [20] to evaluate the overall recovery rates.

### Statistical analysis

The efficiency of cDNA amplification using each primer set was compared in terms of positive rates and Cq values. The positive rates were compared using Cochran’s Q test. The R version 3.1.0 [21] was used for the test. If the null hypothesis, indicating that all positive rates are comparable, was rejected, post-hoc multiple comparisons were conducted using McNemar’s tests with Bonferroni’s adjustment to identify the pairs of assays that were significantly different. The JMP software version 11.1.1 (SAS Institute, Cary, NC) was used for the test. The stool samples were not used in this analysis, because they were first screened using GI-A and GII-A assays, which generate positive rates of 100% for these assays and may bias the test results.

The Cq values were first compared using Friedman’s test. If the null hypothesis, indicating that the Cq values from all assays are comparable, was rejected, post-hoc multiple comparisons were conducted using Scheffe’s test. The JMP software version 11.1.1 (SAS Institute, Cary, NC), with a JMP script provided by SAS Institute Japan (Tokyo, Japan), was used for the test.

Additionally, the relationship between the recovery rates determined with spiked murine norovirus and the Cq values of human norovirus using each assay was tested for the wastewater samples to investigate if the negative results or the high Cq values were because of low recovery rates. Spearman’s rank correlation test with R version 3.1.0 [21] was used for the analysis.

For all tests, statistical significance was set to α = 0.05. To include the negative results in the comparison of Cq values, Cq = 50 was assigned presumptively to those samples. The positive samples with Cq > 40 were considered as “positive but not quantifiable” because the Cq values vary substantially at high Cq and they are not reliable [22], and a presumptive Cq value of 45 was assigned. Since non-parametric tests were used in all analyses, the assigned values (50 and 45) did not affect the test results.

## Results

### Norovirus genotypes and variants in the stool samples

Most noroviruses used in the analysis were genotyped as GII.4 (59/77, 77%). The majority of the strains belonged to Den Haag 2006b variant (36 samples), followed by Sydney 2012 (19 samples) and New Orleans 2009 (4 samples) variants. The other genotypes were GII.2 (2/77, 2.6%), GII.5 (1/77, 1.3%), GII.7 (4/77, 5.2%), GII.14 (6/77, 7.8%), and GI.6 (5/77, 6.5%). The nucleotide sequences were deposited to the International Nucleotide Sequence Database (accession numbers: LC060872–LC060903 and LC074829–LC074873).

### Assay evaluation based on positive rates

The positive rates for wastewater samples were 39–71% with GI assays, and 77–81% with GII assays, respectively (Table 2). For GI assays in wastewater, the lowest positive rate was obtained with GI-B (p = 0.0002, p < 0.0001, and p = 0.0002 vs GI-A, GI-C, and GI-D, respectively). On the other hand, no significant difference was detected between GI-A, GI-C, and GI-D (p > 0.98 for all pairs). For GII assays in wastewater, no significant difference in positive rates was observed among the three assays (p = 0.62). The wastewater samples in which no human norovirus was detected were similar across all the assays tested.

**Table 2:** Number of positive/negative samples and the corresponding positive rates for each assay using wastewater or stool samples.

	Wastewater			Stool		
Assay	Positive	Negative	Positive rate (%)	Positive	Negative	Positive rate (%)
GI-A	48	22	69	– ^a^	– ^a^	– ^a^
GI-B	27	43	39	4	1	80
GI-C	50	20	71	5	0	100
GI-D	48	22	69	5	0	100
GII-A	54	16	77	– ^a^	– ^a^	– ^a^
GII-B	57	13	81	71	1	99
GII-C	54	16	77	71	1	99

^a^ The positive rates were not evaluated because the stool samples were first screened using the GI-A and the GII-A assays to select norovirus-positive stool samples (i.e. positive rates for these assays are theoretically 100%).

### Assay evaluation based on Cq values

**Fig 1:**
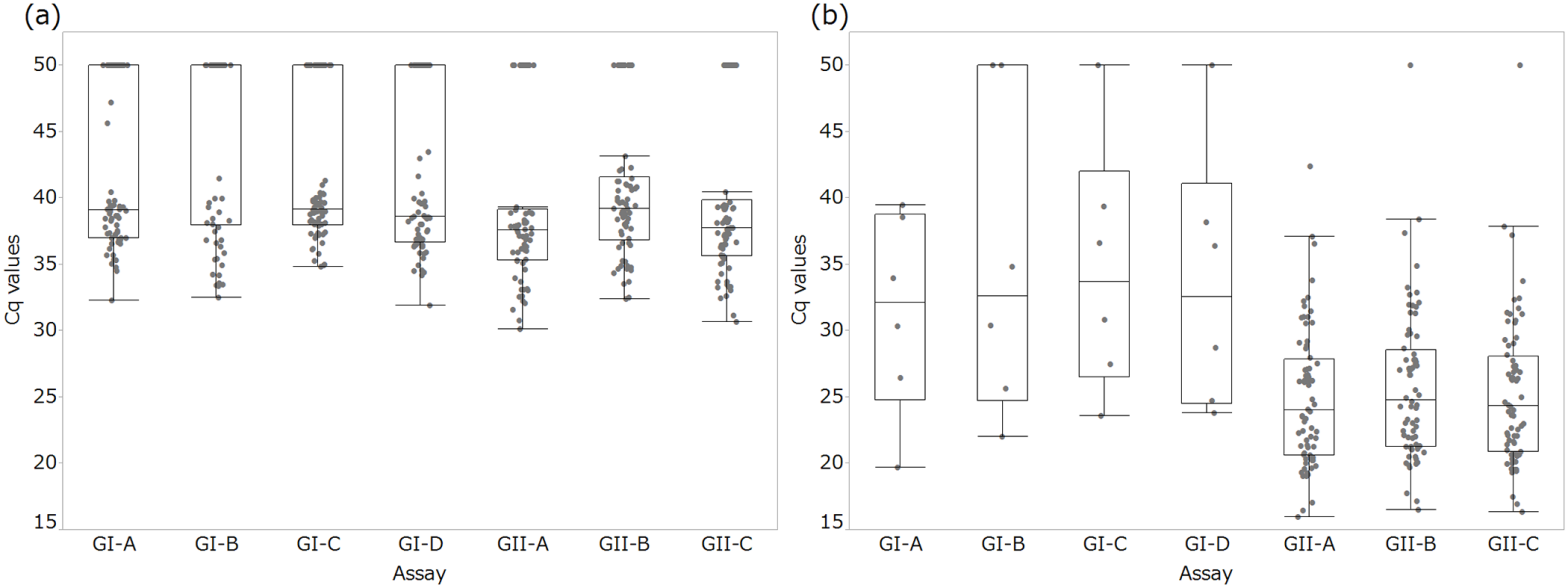
Distribution and box-and-whisker plots of Cq values from qPCR targeting the norovirus GI and GII. The assays were conducted with (a) wastewater samples and (b) stool samples. The box indicates the median and the quartiles. The whiskers are drawn to the furthest points within the 1.5-times interquartile range from the box. Plots at Cq = 45 and 50 show positive but not quantifiable samples (original Cq > 40) and negative samples, respectively.

The distribution of the Cq values obtained with each assay is presented for the wastewater samples (Fig 1a) and the stool samples (Fig 1b). The wastewater samples showed higher Cq values (median Cq > 38 for GI, and 37–39 for GII) than the stool samples (median Cq = 32–33 for GI, and 24–25 for GII), which enabled the evaluation of qPCR assays using a wide range of cDNA concentrations.

For norovirus GI in wastewater, significant differences in Cq values were detected among the four assays (p = 0.0034). The Cq values using GI-D were significantly lower than those using GI-B or GI-C (p = 0.0073 and 0.048); however, the Cq values using GI-A were not significantly different from those using all other GI assays (p = 0.73, 0.97 and 0.14 vs GI-B, GI-C, GI-D, respectively). For norovirus GI in stool samples, there was no significant difference in Cq values between assays (p = 0.28), mostly because of the limited number of samples (n = 6).

For norovirus GII in wastewater, the Cq values with GII-B were significantly higher than those with GII-A or GII-C (p < 0.0001 and p = 0.0002). No significant difference was observed between GII-A and GII-C (p = 0.080). For norovirus GII in stool samples, significant differences in Cq values were detected for all 3 assay pairs (p < 0.0001; all pairs), with this ranking order: GII-A < GII-C < GII-B.

The Cq values from these two sources were combined for analysis. Significant differences were observed in both the GI and GII assays (p = 0.0042 and p < 0.0001). The Cq values obtained using GI-D were significantly lower than those obtained using GI-B or GI-C (p = 0.017 and 0.023). Although the Cq values using GI-A were the second lowest, they were not significantly different from those using all other GI assays (p = 0.69, 0.80 and 0.22 vs GI-B, GI-C, GI-D, respectively). The Cq values obtained using GII-A were significantly lower than those obtained using GII-B or GII-C, and those obtained using GII-C were significantly lower than the Cq values obtained using GII-B (p < 0.0001; all pairs).

**Fig 2:**
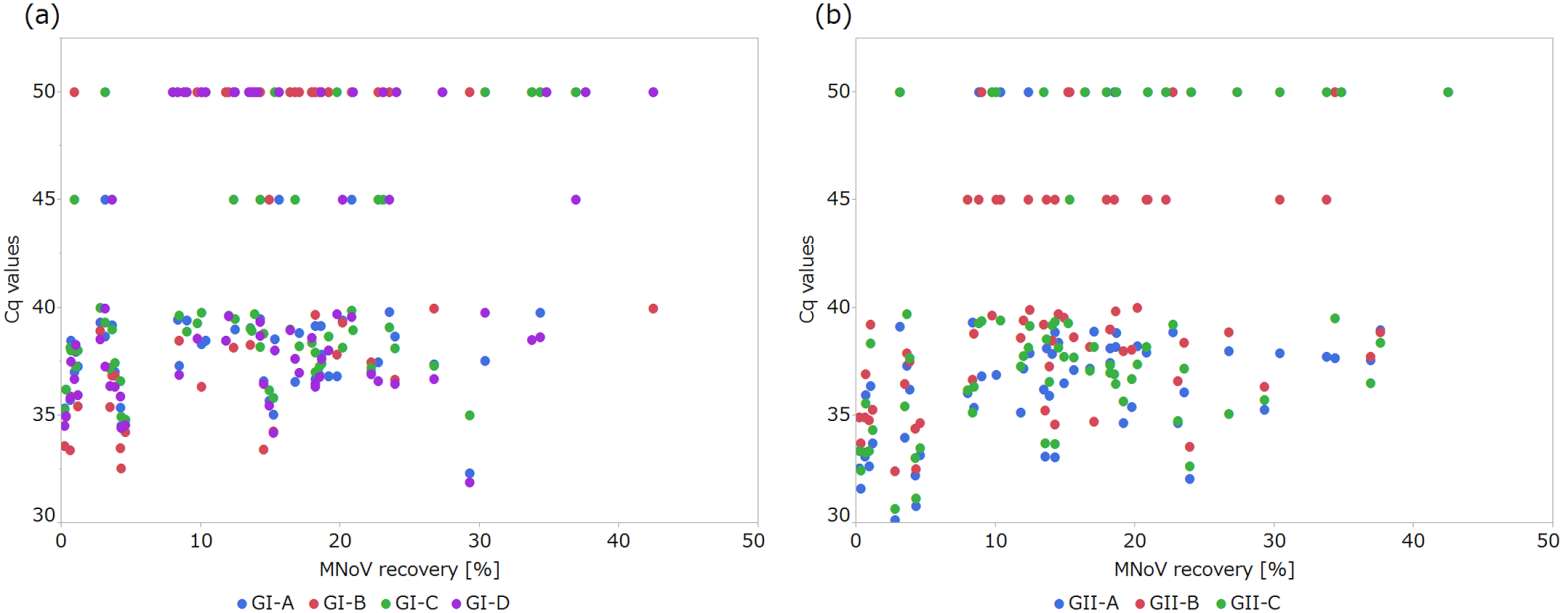
Recovery rates of spiked murine norovirus and Cq values from qPCR targeting the norovirus GI (a) and GII (b). Plots at Cq = 45 and 50 show positive but not quantifiable samples (original Cq > 40) and negative samples, respectively.

The recovery rates of spiked murine norovirus ranged from 0.25% to 43% (geometric mean: 10%). The rank correlation coefficients between the recovery rates and the Cq values of each assay of wastewater samples were 0.22, 0.38, 0.30, and 0.18 for GI-A, GI-B, GI-C, and GI-D; and 0.30, 0.29, and 0.25 for GII-A, GII-B, and GII-C, respectively (Fig 2). Statistically significant correlation were observed for GI-B, GI-C, and all GII assays (p < 0.05).

To investigate if the methods compared in this analysis performed independently of the recovery rates, the wastewater samples were classified into three groups: low (less than 10%, n = 22), middle (10% to 20%, n = 29), and high (more than 20%, n = 19) recovery rates, and the Friedman’s test was performed for each category. Although all category showed significant difference in recovery rates of GI and GII (p < 0.05), the number of pairs that showed significant difference in recovery rates were limited. For GI, only GI-B and GI-D showed significant difference at middle (p = 0.034) and high (p = 0.016) recovery rates. For GII, significant difference was observed between GII-A and GII-B in all categories (p < 0.001, = 0.001 and 0.021 for low, middle and high recovery rates) and GII-B and GII-C in middle recovery rates (p = 0.009). Since all three pairs listed here showed significant difference in recovery rates when all samples were analyzed without classification, the difference in test results between classified and non-classified samples may be because smaller number of samples were used in this analysis with classification.

## Discussion

Molecular assays were developed to detect and quantify enteric viruses in clinical and environmental samples. As there is no cell line to propagate human noroviruses, qPCR is the most frequently used approach for detection. In the present study, clinical and environmental samples were analyzed to compare the positive rates and Cq values of different qPCR methods over a wide range of norovirus genome concentrations. The statistical tests revealed significant differences in both positive rates and Cq values using standardized qPCR assays. Based on the evaluation, GI-D showed significantly smaller Cq values than those of GI-B and GI-C, suggesting that GI-D can detect noroviruses more sensitively than the two methods. It should be noted that since the difference in Cq values between GI-A (2nd lowest in Cq values) and GI-D (lowest Cq values) was not significant and they gave the same positive rate for wastewater, the difference between these two assays is inconclusive. For norovirus GII, GII-A is recommended because while the positive rates were comparable, it yielded significantly lower Cq values than GII-B or GII-C, although some concern remains because GI-A and GII-A assays were used to select norovirus-positive stool samples.

It should be noted that modifications were made from the original methods in the references to evaluate the sensitivity of the qPCR assays (primers/probe design) specifically. In this study, two-step RT-qPCR using identical PCR mastermix (SsoFast probes Supermix, Bio-Rad) and qPCR instrument (CFX96, Bio-Rad) was employed in all assays, while some of the original methods use one-step RT-qPCR [8,10–12] and different reagents and qPCR instruments [8–12]. Although these modifications enabled us to use identical cDNA solution, reagents and instrument in the qPCR analysis, they might cause difference in the sensitivity of the whole method in the references.

Noroviruses are classified into more than 35 genotypes, and outbreaks caused by new norovirus strains are reported frequently. Thus, it is important to update the detection methods constantly to include the emerging genotypes. In this analysis, samples were collected between 2012 and 2013, which included the emerging genotypes and variants, such as the GII.4 Sydney 2012 variant and GI.6. In Japan, the GII.4 Sydney variant was first identified in late 2011, thereby replacing the Den Haag 2006b variant as the major GII.4 variant ever since [23]. The GII.4 variant also predominates in many developed and developing countries [24–30]. The GI.6 was reported as an emerging genotype in the USA in 2010–2012 [31], and it was the only GI genotype detected in the human stool samples. By targeting these emerging genotypes and variants, updated information on the applicability of the existing qPCR assays can be provided. Our results showed that the earliest qPCR methods among tested in this study (GI-A and GII-A) [9] are still valid and sensitive, while the most recent method by Miura *et al*. [8] showed similar performance for GI detection.

The absolute values of recovery rates determined by quantifying murine norovirus spiked into the wastewater samples were low and variable. There was no clear difference in effect of the recovery rates on performance (Cq values) of each assay. All assays showed weak positive correlation between the recovery rates and Cq values, and some assays showed statistically significant correlation. The positive correlation shows that the samples with higher recovery rates contained less amount of cDNA. Thus it is clear that the negative results or high Cq values are not because the recovery rates were low but the concentration of noroviruses in the samples were low.

While this work aimed to evaluate the positive rates and Cq values of the qPCR methods, other aspects should be considered in evaluating standard protocols. For example, it is important to investigate whether the assays are specific to noroviruses. The possibility of false-positive or false-negative should be investigated using various genotypes of noroviruses and other related pathogens and non-pathogens. These analyses should be conducted by multiple laboratory to test the universality of the GI and GII assays using various reagents, qPCR instruments, and sample pretreatment methods, as conducted previously in Canada [32]. Additionally, other parts of the standard methods, such as concentrating samples, RNA extraction, and reverse transcription, were not evaluated in this study. In addition to effectively removing substances inhibiting enzyme activity, concentration methods able to minimize the volume of eluate to submit to RNA extraction would be preferable. These initiatives would enhance understanding of these qPCR assays.
